# Rate of Second Primary Head and Neck Cancer With Cannabis Use

**DOI:** 10.7759/cureus.11483

**Published:** 2020-11-14

**Authors:** Jeehyun Kim, Gordon Hua, Han Zhang, Teffran J Chan, Michael Xie, Marc Levin, Forough Farrokhyar, Stuart D Archibald, Bernard Jackson, James E Young, Michael Gupta

**Affiliations:** 1 Otolaryngology - Head and Neck Surgery, McMaster University, Hamilton, CAN; 2 Otolaryngology - Head and Neck Surgery, University of British Columbia, Hamilton, CAN; 3 Otolaryngology - Head and Neck Surgery, University of Toronto, Hamilton, CAN; 4 Epidemiology and Biostatistics, McMaster University, Hamilton, CAN

**Keywords:** second primary cancer, cannabis, head and neck cancer

## Abstract

Objective

To determine whether there is an association between cannabis use and developing a second primary cancer in head and neck cancer patients, as well as determining the prevalence of cannabis use amongst head and neck cancer patients.

Study design

This retrospective cohort study investigated patients from the Hamilton Region Head and Neck Cancer Database who were enrolled prospectively between 2011 and 2015, with follow-up data up to November 2018. Patients were contacted to confirm current cannabis and tobacco smoking status.

Setting

All patients were enrolled from a single tertiary cancer center in Hamilton, Ontario.

Subjects and methods

Consecutive patients with a newly diagnosed head and neck cancer were prospectively enrolled between 2011 to 2015. Cannabis users and controls were compared using standard modes of comparison. The odds ratio from a multivariable logistic regression model was then determined.

Results

A total of 513 patients were included in this study: 59 in the cannabis group and 454 in the control group. In terms of baseline characteristics, there was no significant difference between cannabis users and controls except that cannabis users were more likely to develop primary oropharyngeal cancer (p=0.0046). Two of 59 (3.4%) cannabis users developed a second primary cancer, in comparison to 23 of 454 (5.1%) non-cannabis users. The odds ratio for cannabis use on the second primary cancer was 0.19 (95% CI [0.01-3.20], p=0.25).

Conclusion

This study suggests that cannabis use behaves differently than tobacco smoking, as the former may not be associated with field cancerization.

## Introduction

Tobacco usage is a well-known risk factor for squamous cell carcinoma of the head and neck (HNC) [1]. This carcinogen leads to potential second primary squamous cell carcinoma (SPC) of the upper aerodigestive tract through the well-studied concept of field cancerization. In field cancerization, the entire aerodigestive tract is exposed to carcinogens causing independent events in all affected tissues, as well as the lateral clonal spread of cancer, which subsequently results in SPC. This concept accounts for the increased risk of SPC in active cigarette smokers following the treatment of HNC [2].

Similar to tobacco smoke, cannabis smoking causes a field exposure to the entire aerodigestive tract and is known to carry similar carcinogens to tobacco with up to twice the concentration of carcinogenic polyaromatic hydrocarbons [3]. Although hypothesized, it is not yet known whether cannabis is a direct causative agent for HNC or increases the risk of SPC through field cancerization. There have been several studies with conflicting results regarding the association between cannabis use and HNC [4-9]. Given that cannabis use is associated with other risky behaviours, there is a possible confounding effect especially when discussing risk for HPV-positive oropharyngeal cancer. Given its anxiolytic and analgesic properties, it may be currently used for symptom management following treatment for HNC without certainty on potential carcinogenic effects. This study explores the risk of SPC with cannabis smoking, which can be used by health care providers in the context of counselling active cannabis users following the treatment of HNC.

Since the legalization of cannabis in Canada on Oct 17, 2018, there has been an increasing self-reported prevalence of cannabis use amongst Canadians with about 5.3 million users over a three-month period in 2019 according to the National Cannabis Survey [10]. This increasing self-reported prevalence may correlate with more cannabis use amongst HNC patients without knowledge of the potential harmful effects. As a result, it is important to examine any possible secondary cancer effects of cannabis use. To our knowledge, this is the first study to examine the potential field cancerization effects of cannabis in HNC patients.

## Materials and methods

Ethics

This study was approved by the Hamilton Integrated Research Ethics Board as database 11-3535.

Patients and chart review

Consecutive patients were enrolled prospectively at the time of biopsy-proven HNC diagnosis into the Hamilton Region Head and Neck Cancer database. All patients were treated at a single tertiary care cancer center and enrolled between January 2011 and January 2015. The database prospectively collected the following characteristics: patient demographics, comorbidities, smoking status (active smoker vs ex-smoker vs never smoker), cannabis use, tumour characteristics, and treatment regimens. A physical review of outpatient, inpatient and cancer clinic records was undertaken to confirm data accuracy and extract relevant patient, tumour, treatment, and follow-up data to determine the second primary status. All patients were followed at tertiary care centers at regular intervals for at least five years. Follow-up data were collected up to November 2018. Patients suspected of SPC were investigated with biopsy and further investigations.

Inclusion and exclusion criteria

Inclusion criteria included patients above the age of 18 years with a pathologically confirmed diagnosis of squamous cell carcinoma of the head and neck enrolled in the Hamilton Region Head and Neck Cancer Database. Exclusion criteria included incomplete data sets, skin cancers and sinonasal tumours. A second primary was defined as a biopsy-proven squamous cell carcinoma of the aerodigestive tract in a separate subsite and location from the primary tumour or a recurrence in the same area greater than five years post-treatment.

Staging

The staging of tumours was clinical and according to the seventh edition of the American Joint Committee on Cancer (AJCC) TNM staging manual (2009).

Telephone follow-up

**Figure 1:**
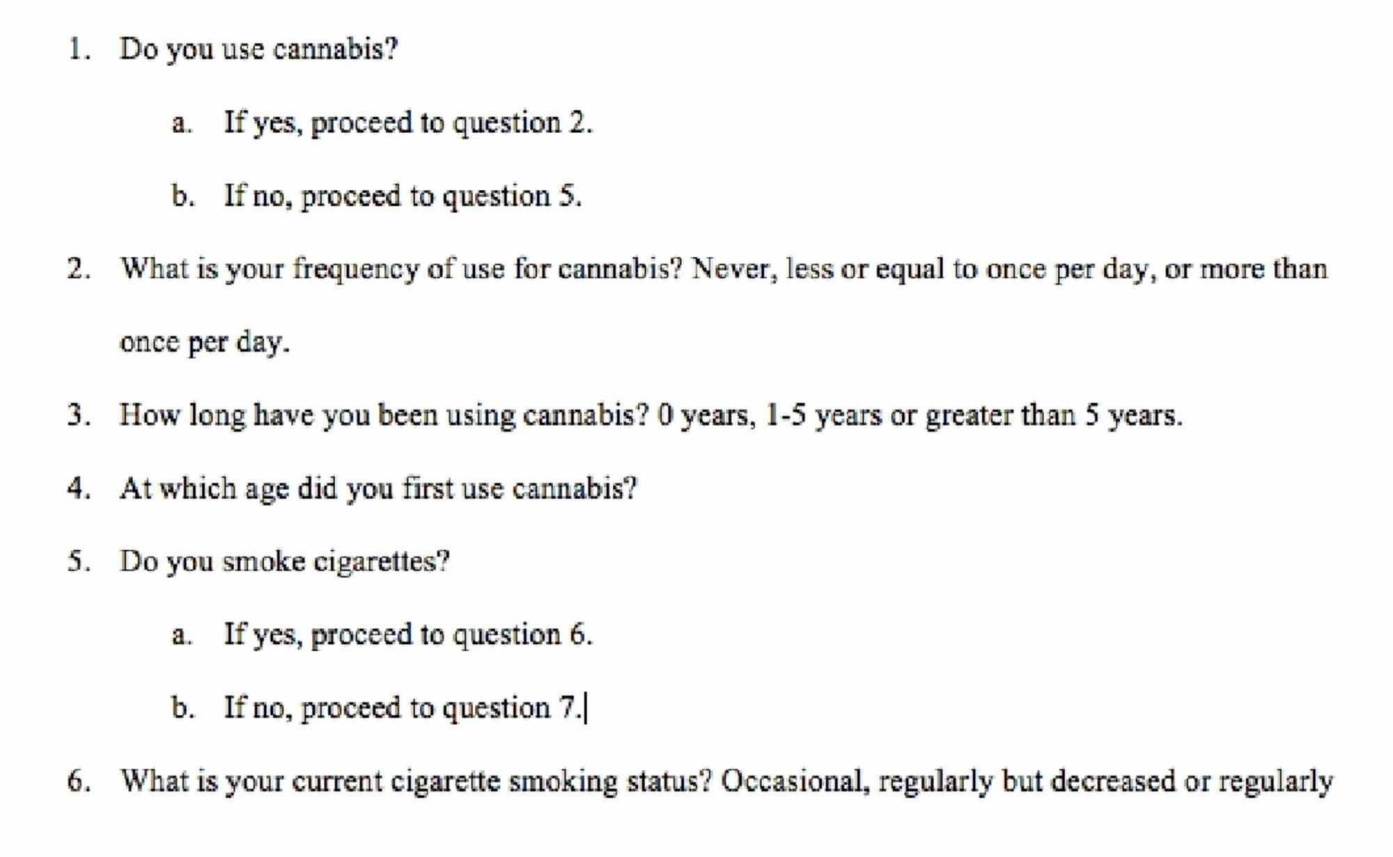
Survey Questionnaire

All patients who were presumed to be alive based on the most recent follow-up date were then contacted via telephone and a further consent process was completed over the phone. This telephone interview determined their current cannabis and cigarette smoking status, frequency of use and duration. The exact telephone questionnaire used is shown in Figure 1. Patients were then grouped based on their most recently identified cannabis and cigarette smoking status. For patients who were unable to be contacted, the original data from the Hamilton Region Head and Neck Cancer Database was presumed to be their most recent cannabis and cigarette smoking status.

Statistical analysis

Patients who were identified as cannabis users were then compared to those who were confirmed as non-cannabis users. The proportion of current cannabis users was identified. Characteristics of age, sex, primary site, primary treatment choice, tumour staging, nodal metastasis staging, cigarette smoking status and alcohol use were summarized. Categorical variables were reported as counts and percentages and compared using the chi-square test or Fisher’s exact test and continuous variables were reported as means with standard deviation or median with range and compared using t-test or the Mann-Whitney U test. The average time to second primary was determined amongst cannabis users, cannabis non-users and the entire cohort. The odds ratio was then determined using the Haldane-Anscombe correction of adding 0.5 to each category [11]. This was used to account for the absence of patients who were cannabis users and tobacco non-users who developed an SPC. Cigarette smokers were excluded from the odds ratio calculation to isolate for cannabis use. P-values less than 0.05 were considered statistically significant.

## Results

Eight-hundred seventy-nine (879) patients were prospectively enrolled in the Hamilton Region Head and Neck Cancer Database. Three-hundred sixty-six (366) patients were excluded based on exclusion criteria, with 67 incomplete charts and 299 diagnoses of exclusion. Of the remaining 513 patients, 66 (12.8%) were identified to be cannabis users and 447 (87.2%) were non-users at the time of enrolment. Following a chart review identifying the most recent follow-up date, 313 patients (60.9%) were presumed to be alive and contacted via telephone. 65 patients (20.7%) completed the survey, 25 (8.0%) refused, three (1.0%) passed away, and 220 (70.3%) were unable to be contacted due to the number not in service, incorrect number on file or the patient did not answer the phone (Figure 1). Of the 65 patients, six (9.2%) identified as active cannabis users and four (6.2) identified as ex-cannabis users. In terms of cigarette smoking status, 11 (16.9%) identified as active cigarette smokers while 29 (44.6%) identified as ex-cigarette smokers.

**Figure 2:**
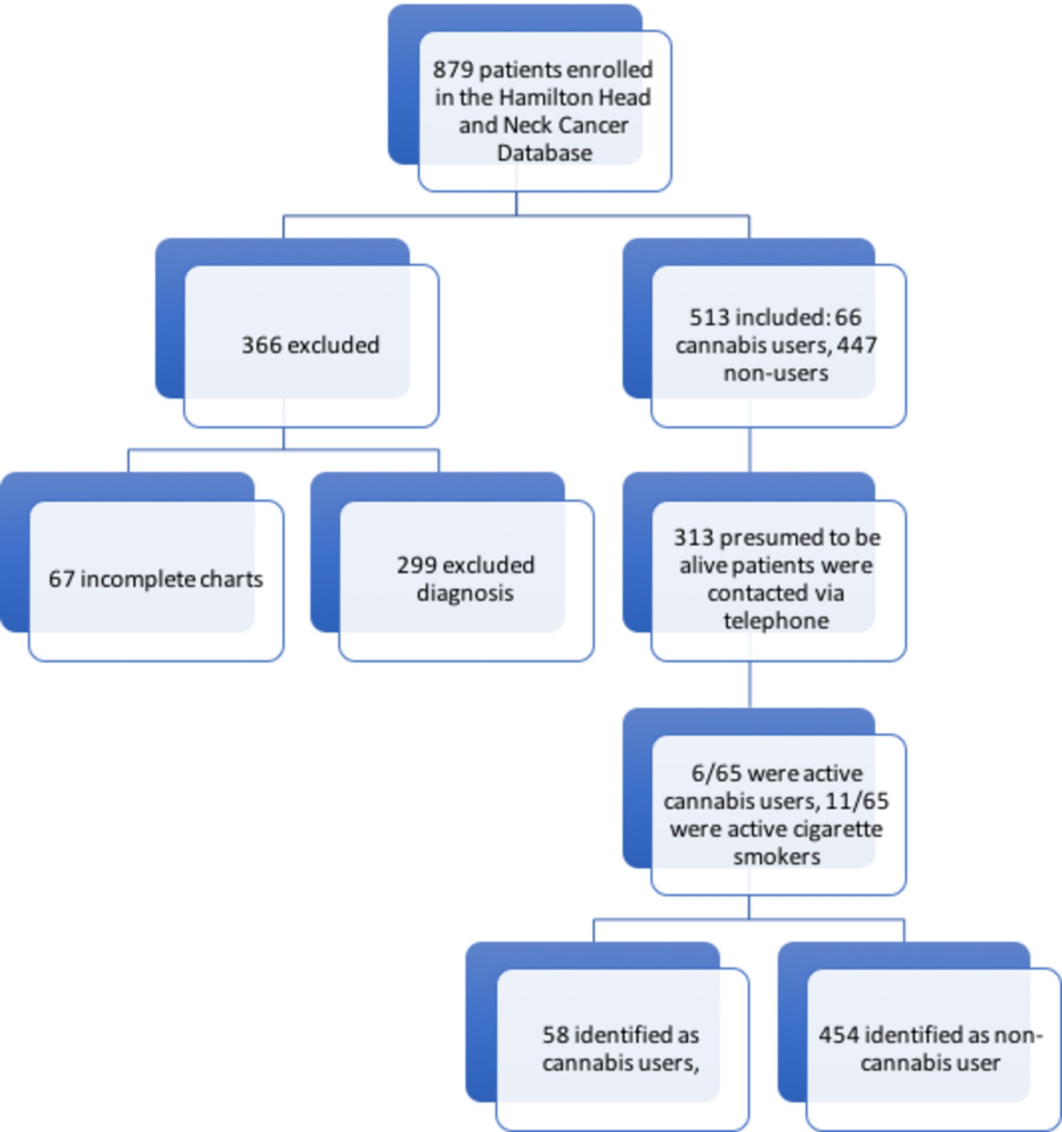
Recruitment Flow Chart

For the final analysis, patients were grouped based on their most recently reported active status for cigarette and cannabis use. There were 59 (11.5%) cannabis users of which 14 were active cigarette smokers and 45 were non-cigarette smokers including ex-cigarette smokers. There were 454 (88.5%) non-cannabis users of which 136 were active cigarette smokers and 319 were non-cigarette smokers including ex-cigarette smokers. Figure 2 shows a flow chart of the process from patient enrolment to survey completion.

Patient characteristics are reported in Table 1. The mean age at diagnosis for the entire cohort was 63 years. 86% of cannabis users were male versus 76% of non-users (p=0.07). Regarding the primary site amongst cannabis users: 61% were oropharyngeal, 13.6% oral cavity, 1.7% nasopharyngeal, and 23.7% laryngeal/hypopharyngeal. Amongst non-cannabis users: 34.8% were oropharyngeal, 29.1% oral cavity, 2.9% nasopharyngeal, 30.4% laryngeal/hypopharyngeal, and 2.9% were unknown primary. There was a statistically significant difference between the groups with cannabis users more likely to develop primary oropharyngeal cancer with a chi-square value of 16.9 with 5 degrees of freedom (p=0.0046). The T status was T1/T2 in 69.4% of cannabis users and 60.6% of non-cannabis users (p=0.19). The N status was N0 in 47.5% of cannabis users as opposed to 52.5% of non-cannabis users (p=0.49). In terms of primary treatment for cannabis users, 22% underwent surgery, 38.9% underwent radiation therapy, 37.3% underwent chemoradiation therapy, and 1.7% were palliative. This finding is in contrast to non-cannabis users where 37.2% underwent surgery, 30.8% underwent radiation therapy, 28.2% underwent chemoradiation therapy, and 3.7% were palliative. The difference between the groups was not statistically significant (p=0.086). In terms of smoking status, 23.7% of cannabis users were active cigarette smokers, 52.5% were ex-cigarette smokers, and 23.7% were never cigarette smokers. This is compared to non-cannabis users where 30% were active cigarette smokers, 48% were ex-smokers and 22% were never cigarette smokers. The differences between the groups were not statistically significant (p=0.61). For alcohol use, 44.1% of cannabis users were also regular alcohol users. This is similar to non-cannabis users, with 45.4% being regular alcohol users (p=0.89). To summarize, there was no significant difference for baseline characteristics between cannabis users and non-cannabis users except that cannabis users were more likely to develop primary oropharyngeal cancer (p=0.0046).

**Table 1:** Patient Baseline Characteristics

	Cannabis User (n = 59)	Control (n = 454)	95% CI	p-value
Mean Age (yr ­± SD)	61.9 ± 10.6	63.6 ± 13.5	-1.93 to 5.32	0.36
Gender				
Male (%)	51 (86.4)	344 (75.8)		0.076
Female (%)	8 (13.6)	110 (24.2)		
Primary site				
Oropharynx (%)	36 (61.0)	158 (34.8)		0.0046
Oral cavity (%)	8 (13.6)	132 (29.1)		
Nasopharynx	1 (1.7)	13 (2.9)		
Hypopharynx (%)	1 (1.7)	14 (3.1)		
Larynx (%)	13 (22.0)	124 (27.3)		
Unknown primary (%)	0 (0)	13 (2.9)		
T status				
T1 / T2 (%)	41 (69.4)	275 (60.6)		0.19
T3 / T4 (%)	18 (30.5)	179 (39.4)		
N status				
N0 (%)	28 (47.5)	237 (52.2)		0.49
N+ (%)	31 (52.5)	217 (47.8)		
Primary treatment				
Surgery	13	169		0.086
Radiation (+Adj)	23 (+6)	140 (+52)		
Chemoradiation (+Adj)	22 (+2)	128 (+23)		
Palliative	1	17		
Social history				
Current cigarette smoker (%)	14 (23.7)	136 (30.0)		0.61
Ex-cigarette smoker (%)	31 (52.5)	218 (48.0)		
Never smoker (%)	14 (23.7)	100 (22.0)		
Rare/Never alcohol (%)	33 (55.9)	248 (54.6)		0.89
Alcohol use (%)	26 (44.1)	206 (45.4)		

Two of 59 cannabis users developed an SPC, and both were also active cigarette smokers. Of the 23 non-cannabis users who developed an SPC, 17 were non-cigarette smokers and six were active cigarette smokers. Eleven of the 17 non-cigarette smokers who developed an SPC were ex-cigarette smokers. The average time to second primary from the initial primary treatment was 72.5 months. When grouped by cannabis use, the average time to the second primary was 85 months for cannabis users and 71.3 months for non-cannabis users. The odds ratio for cannabis use on SPC from a multivariable logistic regression model was 0.19 (95% CI [0.01 to 3.20], p=0.25). These results are summarized in Table 2.

**Table 2:** Odds Ratio Table for Cannabis Use To estimate the odds ratio, the Haldane-Anscombe correction method was used by adding 0.5 to each category. SPC: second primary squamous cell carcinoma

	SPC	No SPC	Odds Ratio (95% CI)	P-value
Cannabis user, cigarette non-smoker	0	45 (100.0%)	0.19 (0.01-3.20)	0.25
Cannabis non-user, cigarette non-smoker	17 (5.3%)	301 (94.7%)		

## Discussion

Slaughter et al. introduced the concept of field cancerization in 1953 when they found histologically abnormal tissue surrounding oral cavity squamous cell carcinoma with multifocal areas of precancerous changes. Field cancerization involves the formation of patches of premalignant disease within the entire surface exposed to carcinogens. This process occurs through genetic and epigenetic alterations replacing normal epithelium with a proliferating field of premalignant cells. For instance, tobacco exposure with cigarette smoking induces precancerous changes along the entire aerodigestive tract with potential for SPC. This theory operates with the understanding that tobacco is a proven carcinogen [12]. As the carcinogenicity of cannabis is not known, research is ongoing regarding its effects and changes to the aerodigestive tract. Therefore, it is still unknown whether this concept of field cancerization can apply to cannabis smoking despite similar exposure along the entire aerodigestive tract. This study was the first to address this gap in knowledge and study the rate of SPC with cannabis use. The results will also help identify the proportion of cannabis use amongst HNC patients.

Our study showed lower odds of developing an SPC with cannabis use with an odds ratio of 0.19, although this result was not statistically significant (p = 0.25). Given that SPC is a rare event, our result was affected by the lack of SPC amongst cannabis users who were non-cigarette smokers requiring the Haldane-Anscombe correction. All 45 patients in this group did not develop an SPC as compared to 17 non-cannabis and non-cigarette smokers who developed an SPC and 301 non-cannabis and non-cigarette smokers who did not develop an SPC. Another important factor to consider is the possible confounding factor of tobacco exposure. The retrospective nature of the initial data collection makes it difficult to quantify the extent of cannabis and cigarette smoking history. Although 17 patients who were not active cannabis or cigarette smokers developed an SPC, 11 of these were ex-cigarette smokers. Additionally, we had a poor response rate in our telephone survey and for patients we were unable to contact, they were presumed to be unchanged from their enrolment cannabis and cigarette smoking status. This may over-estimate the tobacco exposure for smokers that may have quit following the treatment of their primary HNC.

Despite these limitations, our results are consistent with the theory that cannabis is not carcinogenic and hence would not follow patterns of field cancerization. Instead, it is hypothesized that since high-risk behaviours are associated with cannabis use, they may be linked to HNC through the effect of human papillomavirus (HPV) positive disease rather than a true carcinogenic property. This is consistent with the statistically significant higher proportion of primary oropharyngeal squamous cell carcinoma (SCC) within the cannabis users (p=0.0046), which follows the known trend for HPV-positive disease.

Within the literature, research regarding the relationship between HNC and cannabis use has shown conflicting evidence [4-9]. In 1999, Zhang et al. found a strong dose-response pattern of cannabis use on the risk of head and neck cancer although the study recognizes the possible confounding factor of cigarette smoking [4]. Subsequent studies were performed, including the 2004 International Head and Neck Cancer Epidemiology (INHANCE) consortium, which found an inverse association between cannabis and oral cavity SCC. For oropharyngeal SCC, once the confounding effect of HPV was estimated, the association between cannabis with oropharyngeal SCC was almost completely attenuated. Additionally, a separate analysis showed that infrequent cannabis smoking doesn’t confer an increased risk of head and neck SCC [8]. These results are consistent with a recent study from 2019 by Zhang et al. investigating a subset of HPV-positive oropharyngeal SCC within the Hamilton Region Head and Neck Cancer Database. They found no statistically significant survival difference between cannabis users and nonusers [13]. These studies infer a lack of true carcinogenic properties from cannabis use and would support the link between cannabis and HNC to be through risky behaviour instead.

On the other hand, a recent study in 2020 by Liu et al. demonstrated that elevated expression of CNR1 and CNR2 cannabinoid receptors drives the proliferation of existing HPV-positive HNC. This was shown through the activation of the oncogenic p38 MAPK pathway. These findings suggest a potential role of cannabis in the proliferation of HPV-positive HNC growth through an oncogenic pathway as opposed to being solely associated with risky behaviour [14].

Our study determined the prevalence of cannabis use to be 11.4% amongst HNC patients. This proportion is similar to numbers found by Xie et al. although these numbers are lower than the population reported prevalence in Canada [15]. Since cannabis legalization in Canada on October 17, 2018, there has been a trend towards an increase in cannabis use. According to the National Cannabis Survey, the prevalence of cannabis use within Ontario was 14% prior to legalization and 20% in 2019 following legalization [10]. Our study enrolment data, including cannabis use, was collected prior to the legalization, and disclosure rates may therefore be lower.

In a study from 2018, Zhang et al. suggest that cannabis use is associated with significant quality of life benefits, including decreased anxiety, pain, depression, increased appetite and generalized feelings of well-being among patients with newly diagnosed HNC [16]. While our study strengthens the theory that cannabis is a non-carcinogenic entity when used recreationally, further research would be beneficial to study the risk of cannabis use on SPC to allow health care providers to accurately advise patients on the risk of cannabis use following HNC treatment. Especially with the expected rising trend in the prevalence of cannabis use, understanding the effects of cannabis smoking on HNC will become increasingly important for both patients and physicians.

## Conclusions

Our study did not find a significant association between cannabis use and developing an SPC. The results suggest that cannabis behaves differently than tobacco smoking, as it may not be associated with the traditional concepts of field cancerization. Instead, it is hypothesized to behave similarly to HPV-positive disease, as both are linked with high-risk behaviours, although further research is required to establish an association. Our study is the first to attempt to bridge the gap in knowledge regarding SPC rates in association with cannabis use. Further research is warranted to further understand this relationship.
